# The Relationship Between Serum MG53 Levels and the Presence of Metabolic Syndrome and Its Components

**DOI:** 10.3390/medicina61040582

**Published:** 2025-03-25

**Authors:** Serpil Yanık Çolak, Burak Andaç, Eray Özgün, Buket Yılmaz Bülbül, Mine Okur, Ali Cem Yekdeş, Çağla Yıldız, Mehmet Çelik

**Affiliations:** 1 Department of Endocrinology and Metabolism, Trakya University Medical Faculty, Edirne 22000, Turkey; drburakandac87@gmail.com (B.A.);; 2 Department of Biochemistry, Trakya University Medical Faculty, Edirne 22000, Turkey; 3 Department of Public Health, Trakya University Medical Faculty, Edirne 22000, Turkey; 4 Department of Internal Medicine, Trakya University Medical Faculty, Edirne 22000, Turkey

**Keywords:** MG53, metabolic syndrome, insulin resistance, hypercholesterolemia

## Abstract

*Background and Objectives*: MG53 is a myokine/cardiokine involved in membrane repair. Some preclinical studies suggest that it is associated with insulin resistance. Metabolic syndrome (MS) is manifested by dyslipidemia, hypertension (HT), visceral obesity, hyperinsulinism, and glucose intolerance. We aimed to evaluate the relationship between the MG53 protein and MS and its components. *Materials and Methods*: This study was conducted among 64 patients with MS and 64 age- and sex-matched healthy participants. MG53 levels were measured using Human-MG53, a commercially available enzyme-linked immunosorbent assay (ELISA) kit (Cat# CSB-EL024511HU, Alfagen laboratory supplies, Cusabio, Bornova, İzmir.). *Results*: There was no significant connection between serum MG53 levels and the presence of MS (*p* = 0.969). We found no correlation between serum MG53 levels and the presence of HT, weight, waist circumference, body mass index, HDL-C, fasting blood glucose, and HbA1c levels. *Conclusions*: This study’s results suggest no association between serum MG53 levels and MS parameters in the studied ethnic population. Due to the limited number and controversy of available studies on this subject, our findings may provide perspective for conducting studies with more diverse populations to obtain more comprehensive results.

## 1. Introduction

Mitsugumin 53 (MG53), a muscle-specific tripartite motif family protein (TRIM72), is involved in cell membrane repair after acute muscle injury and is expressed in skeletal and cardiac muscle [1,2,3]. Studies have shown that it is found in the lungs (type 1 and 2 alveolar epithelial cells) [4], kidneys (proximal tubular epithelium) [5], and corneal epithelia [6], and is also expressed by macrophages [7]. MG53 consists principally of three functional domains: RING finger and B-box zinc at its N terminus and SPRY domain at its C terminus [1]. MG53 is secreted as a myokine/cardiokine in acute cardiac and skeletal muscle damage and has a protective effect on the organ/tissue [8]. After membrane damage, exposure of the reduced intracellular environment to the oxidized extracellular environment leads to MG53 oligomerization. As a result, intracellular vesicles bound to MG53 are transported to the cell membrane, and local elevation of Ca^2+^ facilitates the fusion of vesicles with the plasma membrane to form a repair patch [1]. In addition, MG53 controls cytosolic calcium through interactions with TRPC4, RyR1, TRPC3, SERCA, CaM1, and Orai1 [9]. Additionally, researchers have demonstrated that MG53 is involved in preconditioning and postconditioning with protective effects on myocardial ischemia/reperfusion (I/R) injury [10,11].

Despite all of these protective effects, chronic elevation of MG53 may cause metabolic disorders [12]. MG53 can function as a ubiquitin E3 ligase through its N-terminal RING domain. E3 ubiquitin ligases directly degrade the insulin receptor, insulin receptor substrate-1 (IRS-1), and other key insulin signaling molecules [13]. As a result, it can cause decreased glucose uptake, hyperglycemia, and insulin resistance. Indirectly, E3 ubiquitin ligases control insulin signaling by managing pro-inflammatory mediators that regulate insulin signaling molecules [14]. According to the pathophysiology described above, some researchers have demonstrated that MG53 may be a pathogenic factor in developing insulin resistance and obesity in animal models and human studies [13,15,16]. On the other hand, certain studies indicated the contrary [17,18,19,20].

Metabolic syndrome (MS) is a disease that starts with insulin resistance, increases the risk of type 2 diabetes mellitus five-fold, and doubles the risk of cardiovascular disease, accompanied by visceral fat accumulation, high blood pressure, fasting hyperglycemia, and abnormal lipid levels [21,22]. Its pathophysiology has not yet been clearly elucidated. Still, it has been shown that increased caloric intake and decreased physical activity, in addition to genetic and epigenetic factors, contribute to the development of MS [23]. Most insulin-induced glucose uptake occurs in skeletal muscle, so insulin resistance in skeletal muscle is likely to predict systemic metabolic dysfunction [24]. According to the pathophysiology described above, it is thought that MG53 acts as a ubiquitin E3 ligase and mediates the degradation of IRS-1, resulting in systemic insulin resistance and ultimately metabolic syndrome [25]. In this context, we aimed to evaluate the relationship between MS and the MG53 protein.

## 2. Materials and Methods

The study design was approved by the Ethics Committee of the University of Medicine of Faculty (TUTF-GOBAEK 2022/344), and patients’ privacy was preserved throughout the study. Individuals whose written informed consent was obtained were included in the study. The study was carried out in accordance with the Declaration of Helsinki.

Power analysis was performed based on similar studies in the literature, and the sample size was determined. When the effect size was calculated as 0.5 based on the study of Hongyang Xie et al. [26], it was calculated that *n* = 64 patients from each group should be included in the study, with a margin of error of 5% and a power value of 80%. Our study included one hundred twenty-eight subjects (*n* = 64 MS; *n* = 64 healthy participants).

The diagnosis of MS was made considering the criteria described below. After the last update in 2009, The International Diabetes Federation (IDF), the American Heart Association (AHA), the National Heart, Lung, and Blood Institute (NHLBI), the World Heart Federation, the International Association for the Study of Obesity, and the International Atherosclerosis Society recommend using the following five criteria, with the presence of any of three qualifying for the diagnosis of MS [27]:

1. Increased waist circumference, with ethnic-specific waist circumference cut-off points (use European data for Eastern Mediterranean and Middle Eastern populations until more specific data are available; Europid populations: males ≥ 94 cm, females ≥ 80 cm).
2. Serum triglycerides ≥ 150 mg/dL or treatment for elevated triglycerides.
3. Serum high-density lipoprotein cholesterol (HDL-C) < 40 mg/dL in males or < 50 mg/dL in females, or treatment for low HDL-C.
4. Systolic blood pressure ≥ 130 mmHg, diastolic blood pressure ≥ 85 mmHg, or treatment for hypertension (HT).
5. Fasting blood glucose (FBG) ≥ 100 mg/dL or previously diagnosed type 2 diabetes.

Individuals without MS, chronic diseases, atherosclerotic cardiovascular disease, obesity (BMI ≥ 30 kg/m^2^), smoking habits, or alcohol consumption were included in the control group. Pregnant women, patients with musculoskeletal disease, those receiving glucocorticoid therapy, those with a diagnosis of malignancy, and those with liver or kidney disease, infection or inflammatory diseases, and decompensated acute disease were excluded from the study in both groups.

Waist circumference, height, and weight measurements were made at the outpatient clinic visit. Body mass index (BMI) was calculated as weight in kilograms divided by height in meters squared. The existence of HT was identified as patients with a mean home and/or office blood pressure reading of 130/85 mmHg or higher or on antihypertensive therapy.

Fasting blood samples were taken from both groups. Serum total cholesterol, LDL-C, HDL-C, triglyceride, FBG, and HbA1c levels were immediately measured. Serum total cholesterol, HDL-C, LDL-C, and triglyceride levels were measured by Roche C702 chemistry analyzer.(Roche Diagnostics, Basel, Switzerland.) In addition, HbA1c was measured by using a Tosoh G8 high-performance liquid chromatography analyzer. Those who received treatment for MS components were also recorded.

The blood samples underwent centrifugation at 1000× *g* for 15 min, separating the serum. The serum samples were frozen at −80 °C so MG53 levels could be measured later. To measure serum MG53 levels, a commercial Human-MG53 enzyme-linked immunosorbent assay (ELISA) kit (Cat# CSB-EL024511HU) was used following the kit’s original protocol. The intra-assay and inter-assay CV% of the kit were <8% and <10%, respectively. We calculated the kit’s detection limit as 8.57 pg/mL by measuring the ten replicates of the zero calibrator (sample diluent). Serum MG53 results were calculated from the standard curve obtained using 1600, 800, 400, 200, 100, 50, 25, and 0 pg/mL standards and expressed as pg/mL. All samples were measured blinded to the patient data. Individuals whose MG53 levels were below the detection limit were excluded from the statistical analysis. The statistical analysis proceeded with the remaining participants.

The categorical variables were presented as numbers (n) and percentages (%). For continuous variables that have a normal distribution, we represented them as mean ± standard deviation. We used the median (25th–75th percentiles) for variables that were not distributed normally. The Shapiro–Wilk test was utilized to evaluate the distribution of all variables. To compare the study groups, we used the independent sample *t*-test to analyze quantitative data with normal distribution. We used the Mann–Whitney U test to analyze quantitative data with a non-normal distribution. We compared the groups using Pearson Chi-square analysis to analyze the qualitative data. Spearman’s rank correlation test was utilized to evaluate the correlation between the variables. *p* values were considered statistically significant if they were less than 0.05. The statistical analysis was performed with the SPSS 22.0 version (IBM Corporation, Armonk, NY, USA). The samples were measured without access to any patient data.

## 3. Results

The two groups, consisting of patients with MS and healthy individuals, were matched in age and gender. Table 1 presents and compares the main clinical and demographic features of the MS group and the control subjects. The MG53 kit’s limit of detection was determined to be 8.57 pg/mL. The serum MG53 levels of 17 patients in the MS group and 24 subjects in the control group were lower than the detection limit. Upon analysis that excluded those below the detection limit, no difference was observed in the MG53 levels between the MS and control groups (*p* = 0.969) (Table 2).

We found no correlation between serum MG53 levels and the presence of HT, weight, waist circumference, body mass index, HDL-C, fasting blood glucose, and HbA1c levels. A weak correlation was found between total cholesterol and serum MG53 levels in the MS group and the entire study population. Table 3 displays the correlation between clinical and laboratory parameters and MG53 serum levels.

The participants were divided into three groups based on their MG53 levels. The group with high MG53 levels had significantly higher triglyceride and total cholesterol levels than those with low MG53 levels (*p* = 0.006, *p* = 0.017, respectively) (Table 4).

## 4. Discussion

MS, which consists of obesity, hyperglycemia, dyslipidemia, and HT components, has increased at an epidemic rate and has become one of the most severe threats to human health. Insulin resistance is the main pathogenic factor for obesity and MS [28]. Insulin resistance in skeletal muscle is likely a critical factor in developing MS, as skeletal muscle is responsible for 70–90% of insulin-stimulated glucose uptake [24].

Song et al. showed that MG53 regulates the insulin signaling pathway in skeletal muscle, and its overexpression leads to insulin resistance [13]. Similarly, Wu and colleagues reported that MG53 is a glucose-sensitive myokine that controls insulin sensitivity via insulin receptors [16]. The study by Song et al. suggests that MG53, which acts as an E3 ligase, might contribute to developing insulin resistance and metabolic disorders [13]. Their research demonstrated sustained increased expression of MG53 in high-fat diet (HFD)-induced obese mice, *db/db* diabetic mice, spontaneously hypertensive rats, non-human primates with MS, and obese humans. In addition, wild-type mice given HFD for 35 weeks with upregulated MG53 were shown to develop MS. According to their study, transgenic mice overexpressing MG53 can develop hyperinsulinemia, hyperglycemia, dyslipidemia, central obesity, and HT, even without an HFD. In the same vein, Wu et al.’s study showed that increases in circulating MG53 were accompanied by increased BMI, hyperglycemia, and hyperinsulinemia in humans and rats with T2DM treated with an HFD (for 35 weeks) [16]. In the same study, researchers compared a small group of obese diabetic humans to a control group of healthy individuals; the results showed that humans with obesity and type 2 diabetes had elevated serum MG53. In another study examining the relationship between serum MG53 levels and impaired glucose tolerance, individuals with normal glucose tolerance have been shown to exhibit reduced levels of MG53 compared to those with impaired glucose regulation [29].

On the other hand, Terauchi et al. [30] and Tamemoto et al. [31] showed evidence of the absence of diabetic phenotypes in IRS-1^-/-^ mice in their study and stated that IRS-1 degradation alone could not trigger the development of diabetes. This is due to the presence of three other homologous proteins (IRS-2, IRS-3, and IRS-4) in the IRS family, each of which has a different physiological role in insulin signal transduction. In particular, IRS-1 and IRS-3 have been shown to serve similar physiological functions in insulin signaling [32]. Since the presence of IRS-3 compensates for the absence of IRS-1, MG53-mediated degradation of IRS-1 is unlikely to cause insulin resistance. In another study evaluating the interaction between IRS-1 and MG53, normal individuals, obese insulin-resistant control subjects without diabetes, and patients with type 2 diabetes before and after insulin infusion were compared, and no difference was found in MG53 interaction with IRS-1 [33]. Similarly, several research groups failed to detect a relationship between insulin resistance/metabolic disorders and the upregulation of MG53 in animals and humans [17,18,19,34,35,36,37,38,39,40]. Our study did not detect a relationship between serum MG53 levels and fasting blood glucose, HbA1c, and clinical parameters related to insulin resistance. However, when interpreting these results, it should be kept in mind that our limited knowledge about the half-life of MG53 in blood circulation may be one of the factors affecting serum MG53 levels. Additionally, the fact that MG53 is an intracellular myokine and that the MG53 level could not be evaluated at the tissue level in our study limits our ability to generalize the results.

Wang et al.’s study [17] showed that insulin signaling and glucose utilization in db/db mice did not change in the case of whole-body ablation of MG53 or sustained elevation of serum MG53. MG53 expression was similar in the skeletal muscles of diabetic animals and healthy controls. However, circulating MG53 was significantly lower in diabetic mice’s blood samples. In this study, 10 diabetic patients and 10 healthy control groups were compared, and no significant correlation was found between FBG levels and serum MG53 levels. To measure serum MG53 levels, they developed a monoclonal antibody against MG53 with high specificity and measured it using the Western blot technique. Although a different technique was used compared to in our study, similar results were obtained. Considering the number of cases in both studies, a more extensive case series should be studied to be able to comment on the preferred measurement technique. Ma et al. [34] examined the expression of MG53 in skeletal and cardiac muscle and serum MG53 levels by comparing mouse models with metabolic disturbances induced by 6-month HFD feeding with mouse models fed a normal diet. Western blotting showed that MG53 expression was not varied in mice with MS in skeletal and cardiac muscles. In addition, it was found that the levels of serum MG53 had decreased. In another study, muscle samples from both diabetic human patients and mice with insulin resistance were found to show normal MG53 expression [38]. Likewise, Yuan et al. [39] and Xu et al. [37] showed that although feeding an HFD caused MS, it did not induce MG53 overexpression. Our study found no relationship between serum MG53 levels and the presence of MS and HT, weight, waist circumference, and BMI; our results are consistent with those of these mentioned studies.

In our study, we compared serum MG53 levels with each parameter of MS. We could not find a significant relationship between serum MG53 levels and the presence of HT, waist circumference, BMI, HDL-C, FBG, and HbA1c levels. However, when the participants were divided into tertiles according to their MG53 levels, triglyceride and total cholesterol levels were significantly higher in the group with high MG53 levels than in the group with low MG53 levels.

Previous studies were mostly designed to examine the relationship between MG53 and insulin resistance and between MG53 and membrane repair. In addition, there are limited studies evaluating the relationship between MG53 and lipid metabolism. However, considering the oxidative stress caused by hypertriglyceridemia and considering that oxidative stress creates cellular stress and activates the protein kinase C (PKC) isoform cascade, the relationship between MG53 and lipid metabolism is intriguing. In this regard, according to a study, mice with high cholesterol and high triglycerides, after being fed an HFD for 8 weeks, showed no changes in MG53 expression in their heart muscle compared to healthy mice. Serum MG53 levels were not evaluated in that study [35]. According to Bai et al., their animal model study showed that oxidative stress occurred in both the hypertriglyceridemia and hypercholesterolemia models; oxidative stress was present in both skeletal muscle and visceral fat in the hypertriglyceridemia group, but it was more severe in the skeletal muscle [41]. It is widely recognized that a rise in reactive molecules leads to the activation of PKC isoforms [42]. Studies have demonstrated that MG53 secretion is mediated by a canonical secretion pathway that requires the activation of H2O2-PKC signaling without cell membrane breakage, and it has been established that MG53 secretion reacts strongly to oxidative stress [8,16]. In addition, another study showed that MG53 might act as a transcription factor and upregulate PPAR-α and target genes, thereby increasing lipid uptake [15]. In our study, triglyceride and total cholesterol levels were significantly higher in the group with high MG53 levels, but we could not detect a correlation between serum MG53 levels and TG levels in the MS group. Although this suggests that there may be patient-based bias, the relationship between triglyceride and total cholesterol levels and serum MG53 level can be evaluated from the perspective we have described above, and this can serve as a starting point for further research on the subject.

Overall, the varying results of the studies can be confusing, but certain factors could contribute to this outcome. It is known that MG53 is a myokine involved in membrane repair in cardiac and skeletal muscle damage. Since MG53 is a myokine, the possibility that its serum levels may be affected by exercise status should be considered. Additionally, depending on the drugs used, glomerular filtration rate, and accompanying tissue damage, serum MG53 levels may vary. The half-life of MG53 in serum and the distribution of MG53 in intracellular and extracellular compartments may also affect the measurements.

## 5. Conclusions

Our findings indicate that circulating levels of MG53 do not differ significantly between individuals diagnosed with MS and healthy controls and provide clinical evidence suggesting an absence of association between MG53 and MS parameters in the particular ethnic group studied. Given that most research in this area is based on preclinical studies and the limited number of existing human studies available, our findings may offer researchers a fresh perspective. They may inspire further investigations involving larger and more diverse populations, ultimately leading to more definitive and impactful insights.

### Study Limitations

It is essential to note some limitations in our study. First, the exercise habits of our subjects may have affected their serum levels of MG53. Although we did not collect data on exercise habits, we only selected individuals who had not exercised heavily and had not received intramuscular injections in the past three days to avoid any potential muscle damage. However, the possible presence of muscle damage could be excluded by measurement of serum creatinine kinase. Additionally, although individuals diagnosed with metabolic syndrome and those with clinical parameters that may be associated with insulin resistance were not included in our control group, the presence of insulin resistance was not completely excluded by the measurement of the HOMA-IR index. Although those with atherosclerotic cardiovascular disease were not included in the control group, it should be kept in mind that there may be individuals who have not yet been diagnosed among the participants. Finally, it is worth considering that MG53 levels may be affected by medication, particularly lipid-lowering treatments.

## Figures and Tables

**Table 1:** The main clinical and demographic features of both groups.

Parameters			Case Group Median (2575 Percentile)	Control Group Median (25–75Percentile)	*p*
Age (/year)			53.50 (45.00–60.00)	51.00 (41.25–57.00)	0.107 ^a^
Gender	Male		25 (39.1%) **	27 (42.2%) **	0.719 ^b^
	Female		39 (60.9%) **	37 (57.8%) **	
Height (/cm)			164.50 (156.00–172.00)	165.00 (160.00–173.75)	0.418 ^a^
Weight (/kg)			92.50 (80.00–104.50)	66.00 (61.00–75.00)	0.001 ^a^
BMI (kg/m^2^)			33.16 (30.48–36.78)	24.42 (23.10–26.97)	0.001 ^a^
WC (/cm)			113.09 ± 11.26 *	85.44 ± 10.20*	0.001 ^c^
Hypertension presence		HT (+)	44 (68.8%) **	-	0.001 ^b^
		HT (−)	20 (31.3%) **	64 (100%) **	
FBG (mg/dL)			107.50 (95.50–126.00)	90.50 (85.00–93.00)	0.001 ^a^
HbA1c (%)			6.35 (6.00–7.27)	5.7 (5.4–6.00)	0.001 ^a^
Triglyceride (mg/dL)			174.50 (114.00–262.25)	100.00 (76.00–148.75)	0.001 ^a^
T. Chol. (mg/dL)			189.50 (160.25–227.00)	193.50 (169.00–221.75)	0.717 ^a^
LDL-C (mg/dL)			115.00 (93.00–146.00)	125.00 (101.25–145.00)	0.232 ^a^
HDL-C (mg/dL)			44.00 (37.00–54.00)	50.50(40.00–62.75)	0.012 ^a^

BMI: body mass index; WC: waist circumference; HT: hypertension; FBG: fasting blood glucose; T.Chol: total cholesterol; LDL-C: low-density lipoprotein cholesterol; HDL-C: high-density lipoprotein cholesterol. * Mean ± SD; ** n (%). ^a^ Mann–Whitney U test. ^b^ Pearson Chi-Square test. ^c^ Independent samples *t*-test.

**Table 2:** Distribution and comparison of parameters in case and control groups (worked by excluding cases with MG53 < 8.57 pg/mL).

Parameters		Case Group MS n = 47	Control Group n = 40	*p*
Gender	Male n (%)	19 (40.4%)	15 (37.5%)	NS ^a^
	Female n (%)	28 (59.6%)	25 (62.5%)	
Age (/year) Mean ± SD		53.6 ± 10.9	50.6 ± 10.3	NS ^b^
BMI (kg/m^2^) Mean ± SD		33.9 ± 4.8	24.6 ± 2.3	0.000 ^b^
WC (/cm) Mean ± SD		112.8 ± 10.7	84.9 ± 10.4	0.000 ^b^
FBG (mg/dL) Med (25.–75. Percentile)		106 (92–126)	90 (84.25–93)	0.000 ^c^
HbA1c (%) Med (25.–75. Percentile)		6.3 (6.0–7.1)	-	-
Triglyceride (mg/dL) Med (25.–75. Percentile)		189 (130–271)	104 (80.5–165.5)	0.000 ^c^
T. Chol. (mg/dL) Mean ± SD		204.6 ± 55.2	201.1 ± 47	NS ^b^
HDL-C (mg/dL) Med (25.–75. Percentile)		44 (37–51)	54 (41.25–61.25)	0.017 ^c^
LDL-C (mg/dL) Mean ± SD		124.3 ± 35.4	130.9 ± 40	NS ^b^
HT presence n (%)		31 (66%)	-	0.000 ^a^
MG53 (0 values excluded) Med (25.–75. Percentile)		82.86 (31.43–141.43)	73.57 (30.71–156.07)	0.969 ^c^

BMI: body mass index; WC: waist circumference; HT: hypertension; FBG: fasting blood glucose; T.Chol: total cholesterol; LDL-C: low-density lipoprotein cholesterol; HDL-C: high-density lipoprotein cholesterol; NS: not significant. ^a^ Pearson Chi-Square test. ^b^ Independent samples *t*-test. ^c^ Mann–Whitney U test.

**Table 3:** Correlation analysis between MG53 levels and parameters in total population, case group, and control group (worked by excluding cases with MG53 < 8.57 pg/mL).

Parameter		Case Group MG53	Control Group MG53	Total Subjects MG53
Age (/year)	r *p*	0.302 0.039	0.071 0.664	0.175 0.105
Height (/cm)	r *p*	−0.064 0.670	−0.054 0.741	−0.066 0.541
Weight (/kg)	r *p*	−0.108 0.470	−0.065 0.689	−0.065 0.553
BMI(kg/m^2^)	r *p*	−0.115 0.440	0.034 0.833	−0.052 0.632
WC (/cm)	r *p*	−0.075 0.617	0.071 0.662	−0.032 0.766
FBG (mg/dL)	r *p*	−0.030 0.839	0.214 0.185	0.016 0.880
HbA1c (%)	r *p*	−0.014 0.926	0.315 0.103	0.026 0.822
Triglyceride (mg/dL)	r *p*	0.109 0.464	0.400 0.011	0.192 0.075
T. Chol. (mg/dL)	r *p*	0.404 0.005	0.138 0.394	0.290 0.006
LDL-C (mg/dL)	r *p*	0.282 0.055	0.051 0.753	0.175 0.105
HDL-C (mg/dL)	r *p*	0.159 0.287	−0.206 0.202	−0.035 0.750

BMI: body mass index; WC: waist circumference; FBG: fasting blood glucose; T.Chol: total cholesterol; LDL-C: low-density lipoprotein cholesterol; HDL-C: high-density lipoprotein cholesterol. r: Spearman’s correlation coefficient.

**Table 4:** Distribution and comparison of parameters according to MG53 tertiles in total population.

			MG53 Tertiles			
Parameters			Low Med (25–75 P.)	Medium Med (25–75 P.)	High Med (25–75 P.)	*p*
Age (/year)			51.00 (41.00–58.00)	52.00 (44.25–55.25)	56.00 (48.00–60.00)	0.172 ^a^
Gender		Male	18 (41.9%) **	19 (45.2%) **	15 (34.9%) **	0.611 ^b^
		Female	25 (58.1%) **	23 (54.8%) **	28 (65.1%) **	
Height (/cm)			165.00 (160.00–175.00)	165.00 (157.75–176.00)	163.00 (156.00–170.00)	0.420 ^a^
Weight (/kg)			75.00 (66.00–88.00)	80.00 (65.75–95.25)	80.00 (69.00–92.00)	0.938 ^a^
BMI (kg/m^2^)			27.50 (24.00–31.60)	29.17 (23.97–33.83)	28.40 (25.00–33.10)	0.730 ^a^
WC (/cm)			97.47 ± 17.39 *	99.86 ± 19.38 *	100.49 ± 15.96 *	0.704 ^c^
HT Pres.	HT (+)		13 (30.2%) **	13 (31.0%) **	18 (41.9%) **	0.446 ^b^
	HT (−)		30 (69.8%) **	29 (69.0%) **	25 (58.1%) **	
DM Pres.	DM (+)		18 (41.9%) **	22 (52.4%) **	24 (55.8%) **	0.403 ^b^
	DM (−)		25 (58.1%) **	20 (47.6%) **	19 (44.2%) **	
FBG (mg/dL)			95.00 (89.00–108.00)	92.50 (87.75–112.50)	93.00 (89.00–107.00)	0.881 ^a^
HbA1c (%)			5.85 (5.55–6.52)	6.10 (5.60–6.85)	6.00 (5.72–6.37)	0.577 ^a^
Triglyceride (mg/dL)			99.00 (85.00–168.00)	117.00 (89.25–195.50)	167.00 (102.00–251.00)	0.006 ^a^
T. Chol. (mg/dL)			187.00 (166.00–210.00)	183.50 (162.75–216.50)	208.00 (182.00–242.00)	0.017 ^a^
LDL-C (mg/dL)			114.00 (93.00–139.00)	117.00 (99.25–143.00)	131.00 (103.00–153.00)	0.194 ^a^
HDL-C (mg/dL)			49.00 (39.00–63.00)	44.00 (38.75–58.00)	47.00 (37.00–57.00)	0.835 ^a^

BMI: body mass index; WC: waist circumference; Pres.: presence; HT: hypertension; FBG: fasting blood glucose; T.Chol: total cholesterol; LDL-C: low-density lipoprotein cholesterol; HDL-C: high-Density lipoprotein cholesterol. * Mean ± SD; ** n (%); ^a^ Kruskal–Wallis test. ^b^ Pearson Chi-Square test. ^c^ Independent samples *t*-test.

## Data Availability

The dataset generated and analyzed during the current study is available from the corresponding author on reasonable request.
